# The impact of *Lactococcus lactis* KUST48 on the transcription profile of *Aeromonas hydrophila*-infected zebrafish spleen

**DOI:** 10.1128/spectrum.03927-23

**Published:** 2024-03-05

**Authors:** Jiayu Chen, Qiuyue Li, Lili Fan, Liqin Xie, Qilin Zhang, Xianyu Deng

**Affiliations:** 1 Department of Modern Agriculture, Zunyi Vocational Technology College, Zunyi, Guizhou, China; 2 Faculty of Life Science and Technology, Kunming University of Science and Technology, Kunming, Yunnan, China; Clemson University, Clemson, South Carolina, USA

**Keywords:** transcriptome, immune response, zebrafish, *Aeromonas hydrophila*, *Lactococcus lactis*

## Abstract

*Aeromonas hydrophila*, an aquatic pathogenic bacterium, has been found to infect many fish species and cause huge aquaculture losses. Antibiotics are the most common drugs used to treat these infections. However, antibiotic abuse can lead to the development of antibiotic resistance. Probiotics have the potential to replace antibiotics for preventing infections. Zebrafish (*Danio rerio*) is a model organism used to study the innate immune system and host-pathogen interactions. Currently, there is little information on how the fish immune system responds to *A. hydrophila* and probiotic treatment. To increase the understanding of the molecular mechanisms behind the zebrafish defense against *A. hydrophila* and provide evidence that antibiotics can be replaced by probiotics, a transcriptome analysis of the zebrafish spleen was conducted 48 hours after infection by *A. hydrophila*, as well as after treatment using *Lactococcus lactis* KUST48 4 hours after infection. A total of 36,499 genes were obtained. There were 3,337 genes found to have significant differential expression between treatment and control groups. According to further annotation and enrichment analysis, differentially expressed genes (DEGs) were involved in signal transduction, endocrine system cancer, and the immune system. Insulin resistance disappeared in the zebrafish after treatment. Quantitative real-time PCR was performed to confirm the significant regulation of immune defense DEGs, the results of which were consistent with the RNA-sequencing data. These results could serve as a basis for future studies on the immune response to *A. hydrophila* and provide suggestions for probiotic alternatives to antibiotics, which will be of great significance to aquaculture and environmental protection.

**IMPORTANCE:**

In recent years, the unreasonable use of antibiotics has led to the emergence of drug-resistant pathogenic bacteria, antibiotic residues, cross infection, toxic side effects, and so on, which has caused a serious threat to human food safety and life health. In recent years, many studies have demonstrated the potential of probiotics as a substitute for antibiotics, but there is still a lack of understanding of the molecular mechanisms underlying probiotic therapy. We conduct a research on the impact of *Lactococcus lactis* KUST48 on the transcription profile of *Aeromonas hydrophila*-infected zebrafish spleen. Mortality of zebrafish infected with *A. hydrophila* was significantly reduced after treatment with *L. lactis* KUST48. Our results can help to strengthen our understanding of the pathogenic mechanisms of zebrafish and provide a valuable reference for the molecular mechanisms of probiotic therapy.

## INTRODUCTION

*Aeromonas hydrophila* is widely distributed in freshwater, marine, and estuarine aquatic environments and is one of the most prevalent human-animal commensal pathogens (1, 2). It can cause intestinal lesions and inflammation in fish (3) causing mass mortality in farmed fish and huge losses in the aquaculture industry (4). It can be effectively and economically treated with antibiotics, but long-term or unwarranted use of antibiotics in feed has led to the development of serious drug-resistant pathogens (5, 6), making prevention and treatment of fish diseases more difficult. Moreover, current studies have mainly examined the mechanism of the disease (4, 7), while few have focused on how to treat infections efficiently and in an environmentally friendly manner.

Probiotics are beneficial microorganisms that can survive in the host body and colonize the intestinal tract and reproductive system (8). Many probiotics have been shown to inhibit the growth of pathogenic bacteria and provide benefits to the host (9–12). Treatment with probiotics does not leave drug residues or lead to the evolution of antibiotic resistance, which can be harmful to animal and environmental health (13). *Lactococcus lactis* is a probiotic that promotes growth and immune regulation (14). It has been shown that the microbial exopolysaccharides it produces can significantly enhance the non-specific immune response, cell proliferation, and phagocytic activity (15).

High-throughput sequencing technology has been applied for gene expression analysis, differential expression analysis, mining of novel genes, and gene function annotation; it now plays an important role in biological research (16). In aquaculture, the immune mechanisms of many fish and shrimp have been further elucidated by transcriptome sequencing in animals such as goldfish (*Carassius auratus* L.) (17), red swamp crayfish (*Procambarus clarkii*) (18), *Fenneropenaeus merguiensis* (19), northern snakehead (*Channa argus*) (20), and zebrafish (*Danio rerio*) (21).

In recent years, zebrafish (*D. rerio*) have become an important tool for immune disease research (1, 22) because of their small size, short generation time, high reproductive output, rapid external development, and optical transparency (4). The spleen is an important immune organ that is capable of recognizing and clearing pathogens in the body, thereby protecting the body from infection (23, 24). It can produce blood, produce antibodies, and filter blood (25). In this study, transcriptome analysis of *A. hydrophila*-infected and *L. lactis-*treated zebrafish was performed. The immune mechanism of the zebrafish spleen and the effect of *L. lactis* on the immune response were revealed. The results of this study will provide further insight into the pathogenesis of *A. hydrophila* and provide a reference for the mechanism of *L. lactis* inhibition of *A. hydrophila*. This study indicates that probiotics are a real alternative to antibiotic treatment, which is of great value to the aquaculture industry and environmental protection.

## MATERIALS AND METHODS

### Antibacterial experiment *in vitro*

The *A. hydrophila* strain GDMCC 801075 was obtained from Guangdong Microbial Culture Collection Center, and the *L. lactis* KUST48 strain was isolated from tilapia fish (12). To prepare the semi-solid media, 5 mL of Luria-Bertani (LB) was mixed with 100 µL of 10^6^ CFU/mL *A*. *hydrophila* and thoroughly mixed. The mixture was then spread onto solid LB plates before solidification. Sterilized Oxford cups were placed on the solidified media in LB plates. Next, 100 µL of cell-free supernatant from strain *L. lactis* KUST48 was added into the Oxford cups, while kanamycin and ddH_2_O of the same volume served as positive and negative controls (CTs), respectively. After being placed in the refrigerator at 4°C for 4 hours, the plates were then cultured at 37°C. The diameter of the inhibitory zone was measured after 48 hours.

### Fish treatment, bacteria, and sampling

Zebrafish (mean length: 3.74 ± 0.23 cm) of the same developmental period were obtained from the China Zebrafish Resource Center. They were kept in a tank with flowing water at 28°C. The day-to-night ratio was set to 14:10. Zebrafish were cultured at 28°C for 7 days and randomly divided into three groups, each with 60 individuals. Three replicates were set up for each group (20 individuals in each replicate). Zebrafish fasted for 24 hours before injection.

In the *A. hydrophila* infection (AHI) and *L. lactis* KUST48 treatment (LLT) groups, 10 µL of 10^7^ CFU/mL *A*. *hydrophila* was injected intraperitoneally into zebrafish; the CT group fish were injected with 10-µL phosphate-buffered saline (PBS). Additionally, 10 µL of 10^8^ CFU/mL *L*. *lactis* KUST48 was injected into the LLT group zebrafish 4 hours later, while 10-µL PBS was injected into the CT and AHI zebrafish. Nine fish from each group (three fish per replicate) were randomly selected for spleen tissue collection at 48 hours after challenge. Three fish spleens from each group were randomly collected for histopathological examination according to a standard protocol (26). Briefly, the spleens were fixed in 4% paraformaldehyde, dehydrated, and embedded in paraffin. The embedded spleens were then sectioned and stained with hematoxylin and eosin (H&E). Animal experiments were approved by the Ethical Committee of Kunming University of Science and Technology.

### Enzyme activity detection

The activity of lysozyme (LZM), alkaline phosphatase (AKP), acid phosphatase (ACP), and superoxide dismutase (SOD) was measured using enzyme kits (Nanjing Jiancheng Bioengineering Institute, China). The enzyme activity results were expressed as a unit of enzyme activity per milligram of protein.

### Total RNA extraction, cDNA library construction, and sequencing

Total RNA was isolated from spleens from AHI, LLT, and CT zebrafish using TRIzol (Invitrogen). A TruSeq RNA Sample Preparation Kit (Illumina) was used to construct transcriptome library. The mRNA was isolated using oligo (dT) beads and subsequently fragmented into small pieces. Double-stranded cDNA was synthesized using a SuperScript double-stranded cDNA synthesis kit (Invitrogen), and then, the synthesized cDNA was subjected to end-repair and phosphorylation. Sizes of 300-bp cDNA were selected from the agarose gel after 15 PCR cycles. The paired-end RNA-sequencing (RNA-seq) library was sequenced with the Illumina NovaSeq 6000 sequencer platform at Majorbio Genome Center (Shanghai, China).

### Transcriptome data analysis, assembly, and annotation

SeqPrep and Sickle were used to remove reads containing adapters and reads failing quality inspection with default parameters. Clean reads were obtained and aligned to the reference genome using HISAT2 (27). StringTie was used to assemble the filtered data (28). Annotation of gene functions was performed based on the six following common databases: Pfam, EggNOG, Nr, KEGG, Swiss-Prot, and GO.

### Selection of DEGs and enrichment analysis

The gene expression levels were estimated by RSEM (RNA-Seq by Expectation Maximization) software (29). The differentially expressed genes (DEGs) among different groups were obtained with DESeq2 (30) v1.22.2 Bioconductor R package, and gene sets with |log2FC| > 2 and *P*-value < 0.05 were selected. KEGG pathway analysis and GO functional enrichment were conducted using Goatools and KEGG Orthology-Based Annotation System (31).

### qRT-PCR validation

To validate the data of sequences, we randomly selected 20 genes from ordinary DEGs for quantitative real-time PCR (qRT-PCR). Primers were designed by PrimerQuest Tool. Ribosomal protein L13 (*rpl13*) was used as a CT gene. qPCR reactions were performed following the manufacturer’s protocol of the SYBR Premix Ex Taq II Kit (Takara Bio). The cycling conditions were 95°C for 1 min, followed by 40 cycles of 95°C for 15 s and 60°C for 30 s. Three technical replicates and triplicate biological experiments were performed. The 2^ΔΔ-CT^ method was used to analyze and process data (32).

## RESULTS

### Antibacterial effects of *L. lactis* KUST48

Negative CT group showed no antibacterial zone. The positive CT group showed an inhibitory zone with an average diameter of 17.93 ± 0.58 mm. The inhibitory zone diameter (10.97 ± 0.46 mm) of *L. lactis* KUST48 was significantly larger than that of the negative CT group (*P* < 0.001). These results indicate that the cell-free supernatant from strain *L. lactis* KUST48 has a significant inhibitory effect on the growth of *A. hydrophila*.

### Morphology and histopathology

**Fig 1:**
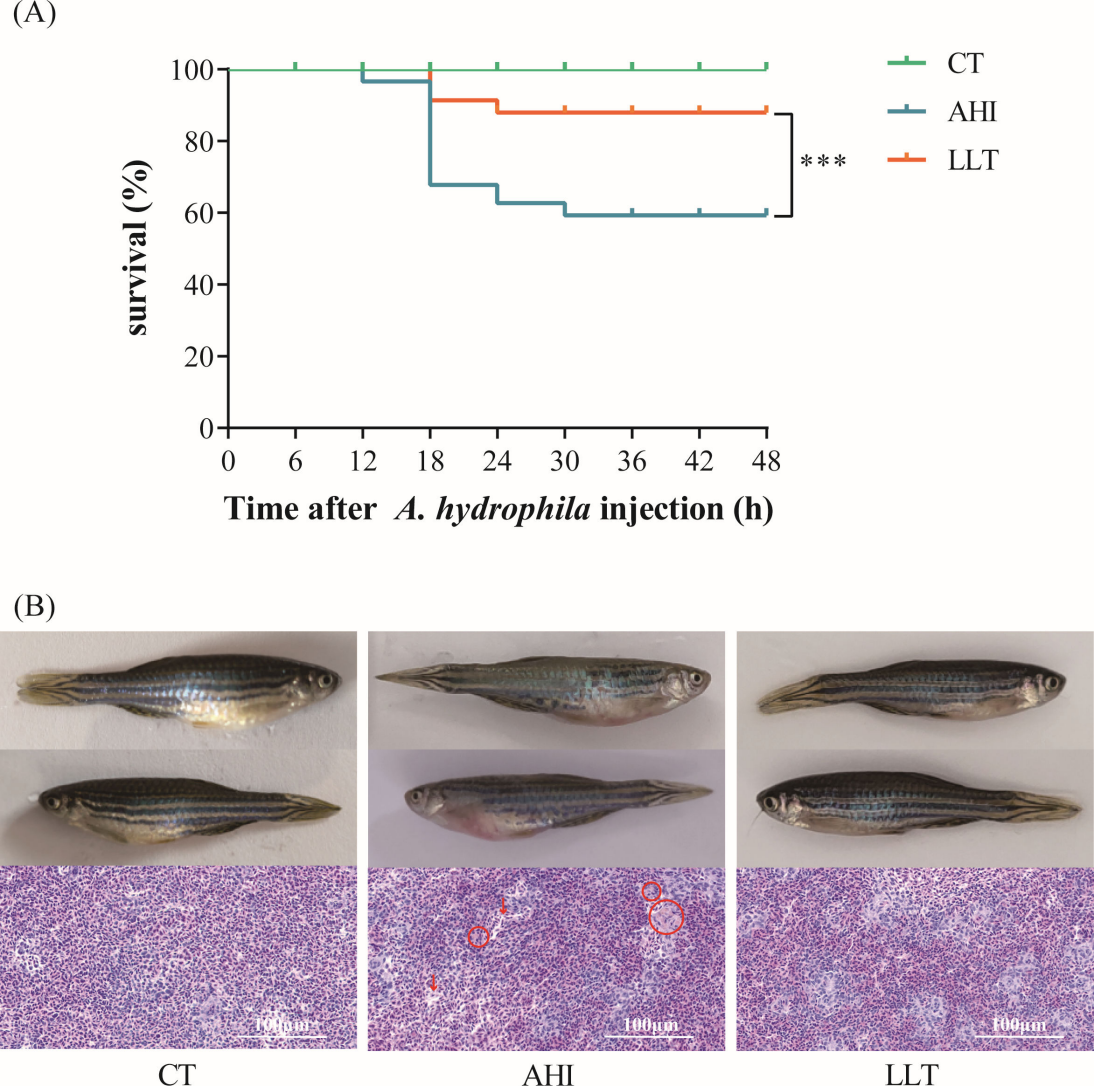
The effect of *A. hydrophila* on zebrafish. (**A**) Survival of zebrafish in different group. (**B**) Representative morphology and spleen H&E staining of zebrafish. Tissue relaxation (red arrow) and congestion were observed in zebrafish spleen from the AHI group as well as increased number of macrophages and hemosiderin (red circle).

The survival of the fish was observed every 6 hours after injection with *A. hydrophila*. The survival rate of zebrafish in the LLT group was significantly higher than that of the AHI group (Fig. 1A). Zebrafish in the AHI group showed abdominal swelling and bleeding, and zebrafish in the CT group and LLT group were asymptomatic (Fig. 1B). The histological changes in *A. hydrophila*-infected spleen were compared with healthy zebrafish. Hematogenous red pulp and compact structure were visible in the spleen of the CT group fish. However, tissue relaxation (red arrow) and congestion were observed in zebrafish spleen from the AHI group as well as an increased number of macrophages and hemosiderin (red circle). The spleen structure of zebrafish in the LLT group is compact with mild congestion. Also, the increased number of macrophages in the spleen was observed (Fig. 1B).

### Enzyme activity

**Fig 2:**
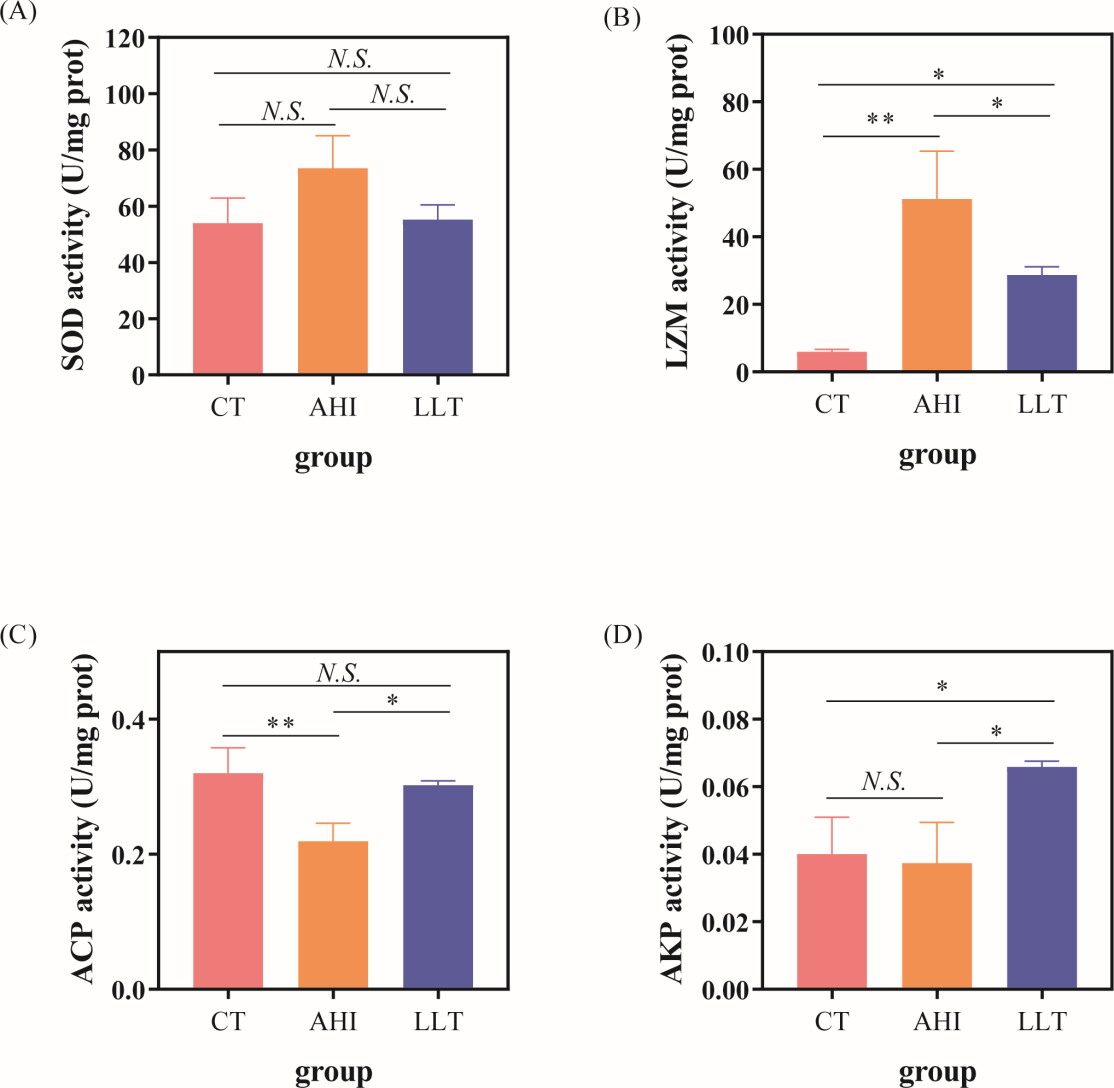
Activity of enzyme in the spleen of *A. hydrophila*-infected zebrafish at 48 hours. (**A**) SOD activity. (**B**) LZM activity. (**C**) AKP activity. (**D**) ACP activity. N.S. means no significance; *P < 0.05; **P < 0.01.

Immune-related enzyme activities were detected to further understand the immune regulatory mechanisms. Compared to the AHI group, the enzyme activity of the LLT group is more similar to the CT group. The SOD (Fig. 2A) and LZM (Fig. 2B) activities of the AHI group were higher than those of the other two groups. However, ACP (Fig. 2C) activity of the AHI group was significantly lower than that of the other two groups. The ACP (Fig. 2D) activity of the LLT group was significantly higher than that of the other two groups.

### Summary of RNA-seq data

After removing unqualified reads, CT groups (CT 1, CT 2, and CT 3) provided 40.70, 45.59, and 44.11 million clean reads, respectively; AHI groups (AHI 1, AHI 2, and AHI 3) provided 49.87, 46.31, and 52.23 million clean reads, respectively, while LLT groups (LLT 1, LLT 2, and LLT 3) provided 50.81, 48.48, and 57.00 million clean reads, respectively (Table 1). A total of 435,125,914 reads were obtained from the AHI, LLT, and CT groups. There were 74,025 transcripts, including 47,628 annotated transcripts and 26,397 new transcripts after assembly; the transcripts were clustered into 36,499 genes. The clean reads for each sample were matched against the reference genome; the mapping rate was 88.09%–91.26%. Six databases were selected for comparison with assembled genes: GO, KEGG, COG, NR, Swiss-Prot, and Pfam. It was found that 26,221 (95.23%) genes were annotated by at least one database, containing 22,346 (81.16%) in GO, 16,964 (61.61%) in KEGG, 25,242 (91.68%) in COG, 26,091 (94.76%) in NR, 23,488(85.31%) in Swiss-Prot, and 22,744 (82.61%) in Pfam (Table 2).

**TABLE 1:** Summary of high-quality sequencing reads

Sample	Raw reads	Clean reads	Total mapped	Multiple mapped	Uniquely mapped
LLT1	51,221,552	50,805,786	45,900,219 (90.34%)	7,735,893 (15.23%)	38,164,326 (75.12%)
LLT2	48,898,416	48,483,116	43,727,129 (90.19%)	7,607,052 (15.69%)	36,120,077 (74.5%)
LLT3	57,447,352	57,001,302	51,528,994 (90.4%)	9,236,406 (16.2%)	42,292,588 (74.2%)
AHI1	50,316,478	49,869,776	45,339,153 (90.92%)	7,425,294 (14.89%)	37,913,859 (76.03%)
AHI2	46,828,458	46,313,702	42,265,836 (91.26%)	11,305,258 (24.41%)	30,960,578 (66.85%)
AHI3	52,703,762	52,237,100	47,184,274 (90.33%)	7,789,521 (14.91%)	39,394,753 (75.42%)
CT1	41,183,372	40,706,144	36,789,071 (90.38%)	5,988,185 (14.71%)	30,800,886 (75.67%)
CT2	46,069,044	45,595,208	40,757,977 (89.39%)	5,410,551 (11.87%)	35,347,426 (77.52%)
CT3	44,882,776	44,113,780	38,859,564 (88.09%)	6,262,363 (14.2%)	32,597,201 (73.89%)

**TABLE 2:** Functional annotation of assembled genes

Database	Number of genes	Percentage
GO	22,346	81.16%
KEGG	16,964	61.61%
COG	25,242	91.68%
NR	26,091	94.76%
Swiss-Prot	23,488	85.31%
Pfam	22,744	82.61%
Total annotated	26,221	95.23%
Total	27,533	100.00%

### Analysis of DEGs

**Fig 3:**
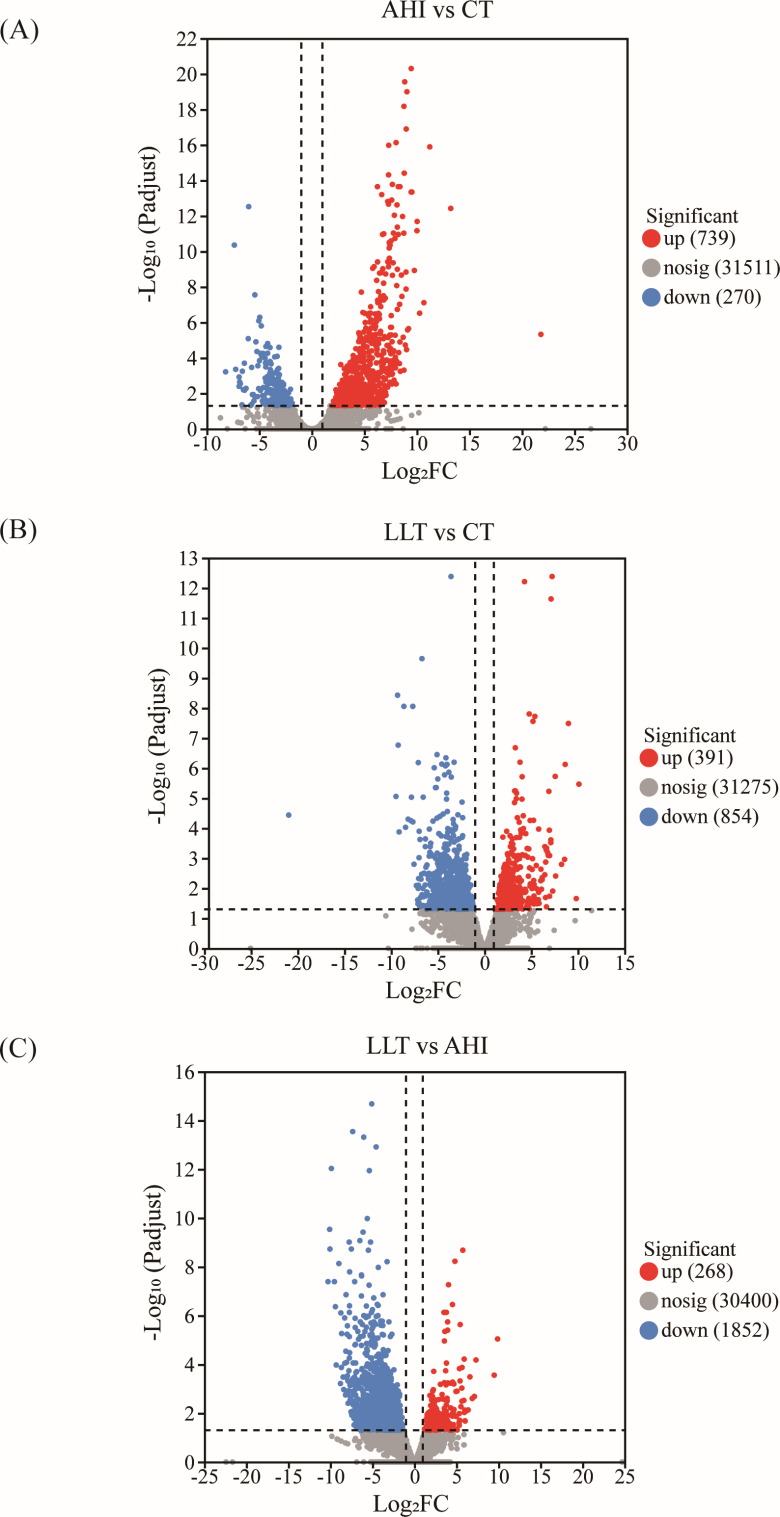
Volcano plot of differently expressed genes. (**A**) AHI vs CT. (**B**) LLT vs CT. (**C**) LLT vs AHI.

The number of DEGs among the different groups (AHI vs CT, LLT vs CT, and LLT vs AHI) was compared. A total of 3,337 DEGs were obtained in the spleen tissue. Specifically, 1,009 DEGs were obtained in the AHI vs CT group, with 739 upregulated genes and 270 downregulated genes (Fig. 3A). In the LLT vs CT group, there were 1,245 DEGs, including 391 upregulated genes and 854 downregulated genes (Fig. 3B). In the LLT vs AHI group, 2,120 DEGs were obtained, including 268 upregulated DEGs and 1,852 downregulated DEGs (Fig. 3C).

### GO functional classification of DEGs

**Fig 4:**
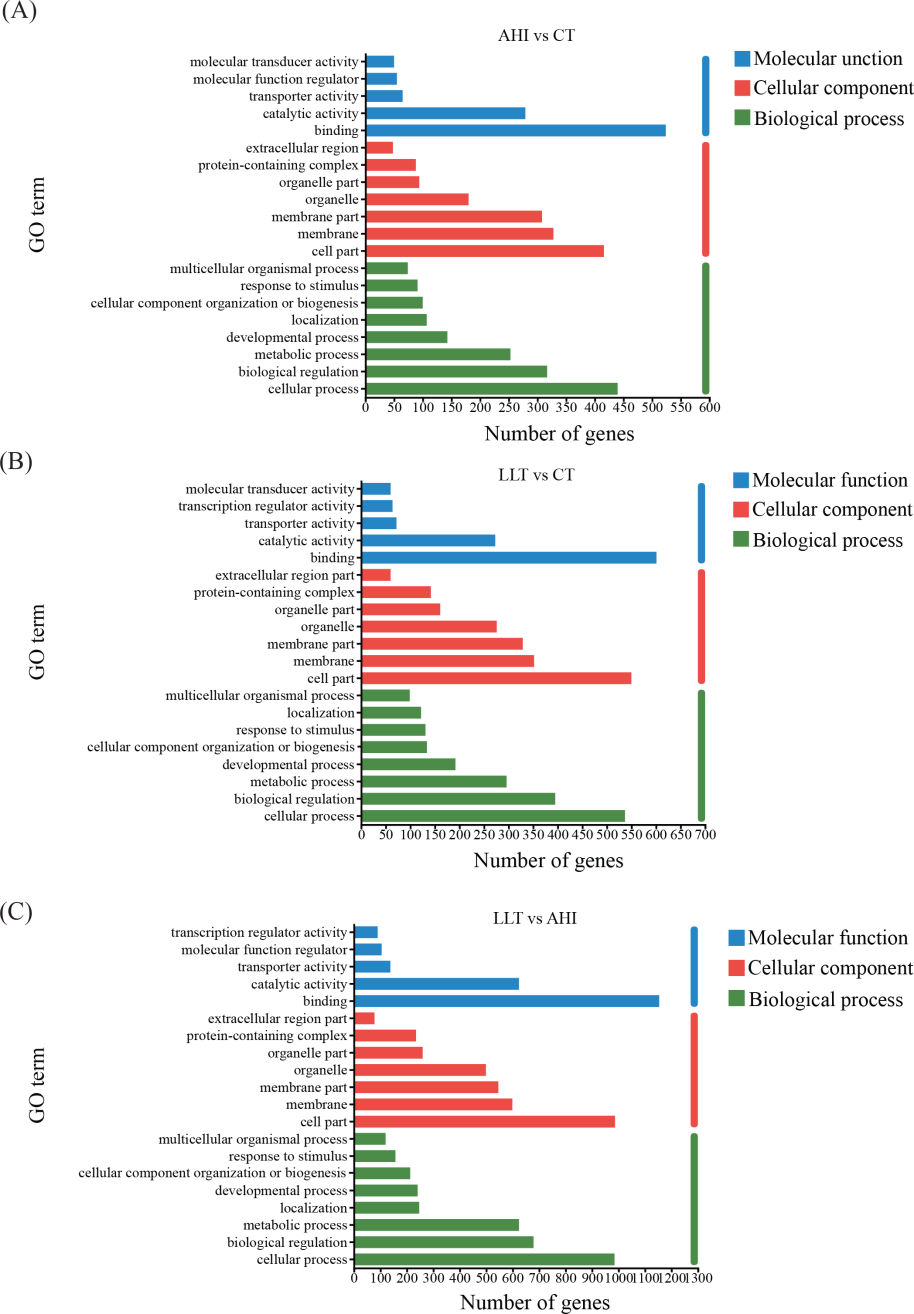
GO classifications of DEGs among different groups. DEGs were annotated into three GO categories: biological process, MF, and CC. (**A**) AHI vs CT. (**B**) LLT vs CT. (**C**) LLT vs AHI.

Functional classes of single genes were identified by GO and KEGG (Table S1). GO functional annotation classification includes three parts: cellular components (CCs), molecular functions (MFs), and biological pathways (BPs). There were 1,009 DEGs in the AHI vs CT group. Most of the genes in the CC were involved in cell and membrane components. The BP category primarily includes aspects of cellular processes, biological regulation, and metabolic processes. Within the MF category, binding, catalytic activity, and transporter activity are highly represented (Fig. 4A). There were 1,245 DEGs in the LLT vs CT group. Most DEGs participated in binding (601 DEGs), cell parts (550 DEGs), cellular process (537 DEGs), biological regulation (395 DEGs), membranes (352 DEGs), membrane parts (329 DEGs), metabolic processes (296 DEGs), and organelles (276 DEGs) (Fig. 4B). A total of 2,120 DEGs were annotated in the LLT vs AHI group and assigned to 20 functional terms from the GO database. Among them, the most enriched DEG in BP was the cellular process subcategory (985 DEGs, 46.5%), followed by biological regulation (679 DEGs, 32.0%) and metabolic processes (624 DEGs, 29.4%). Under the domain of CCs, the top three categories were cell parts (987 DEGs, 46.6%), membranes (599 DEGs, 28.3%), and membrane parts (546 DEGs, 25.8%). In terms of MF, DEGs were mainly involved in binding (1154 DEGs, 54.4%), catalytic activity (624 DEGs, 29.4%), and transporter activity (138 DEGs, 6.5%) (Fig. 4C).

### KEGG functional annotation and classification of DEGs

**Fig 5:**
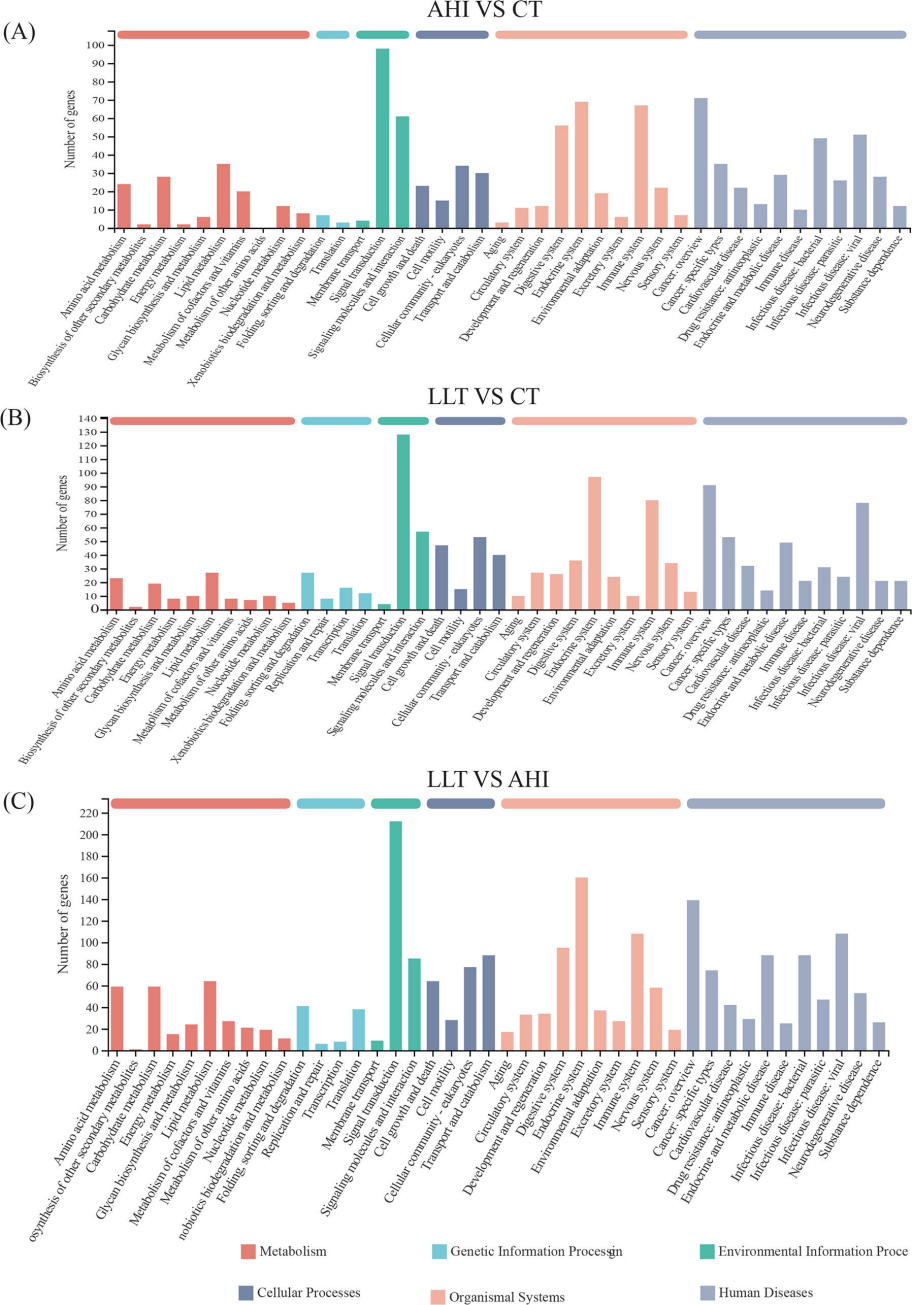
KEGG annotation of DEGs among different groups. Different colored bars represent different groups. (**A**) AHI vs CT. (**B**) LLT vs CT. (**C**) LLT vs AHI.

KEGG pathway enrichment analysis was performed on 3,337 DEGs and annotated into six major groups, including metabolism, organismal systems, genetic information processing, environmental information processing, cellular processes, and human diseases (Table S2). The first four prevalent pathways in the DEG enrichment in all groups were signal transduction, endocrine system, cancer: overview, and immune system. More specifically, DEGs in the AHI vs CT group were mapped to 40 KEGG pathways. In environmental information processing, 98 DEGs and 61 DEGs were assigned to signal transduction and signaling molecules and interaction, respectively. In organismal systems, 69 DEGs and 67 DEGs were assigned to the endocrine and immune systems, respectively. There were 71 DEGs belonging to cancer: overview under human diseases (Fig. 5A). Genes in the LLT vs CT group were assigned to 42 pathways. These pathways were mainly related to signal transduction, endocrine system, cancer: overview, and immune system (Fig. 5B). DEGs in the LLT vs AHI group were mapped to 42 KEGG pathways. Among these, signal transduction had the most DEGs (212), followed by the endocrine system (160 DEGs) and cancer: overview (139 DEGs) (Fig. 5C).

### Enriched KEGG pathways

DEGs in all groups were mapped to the KEGG database. A total of 630 DEGs in the AHI vs CT group were divided into 281 pathways. There were 20 immune system-related pathways identified. The top five pathways were hematopoietic cell lineage (13 genes, 2.06%), chemokine signaling pathway (11 genes, 1.74%), complement and coagulation cascades (10 genes, 1.59%), leukocyte transendothelial migration (nine genes, 1.43%), and interleukin (IL)-17 signaling pathway (eight genes, 1.27%) (Table S3).

**Fig 6:**
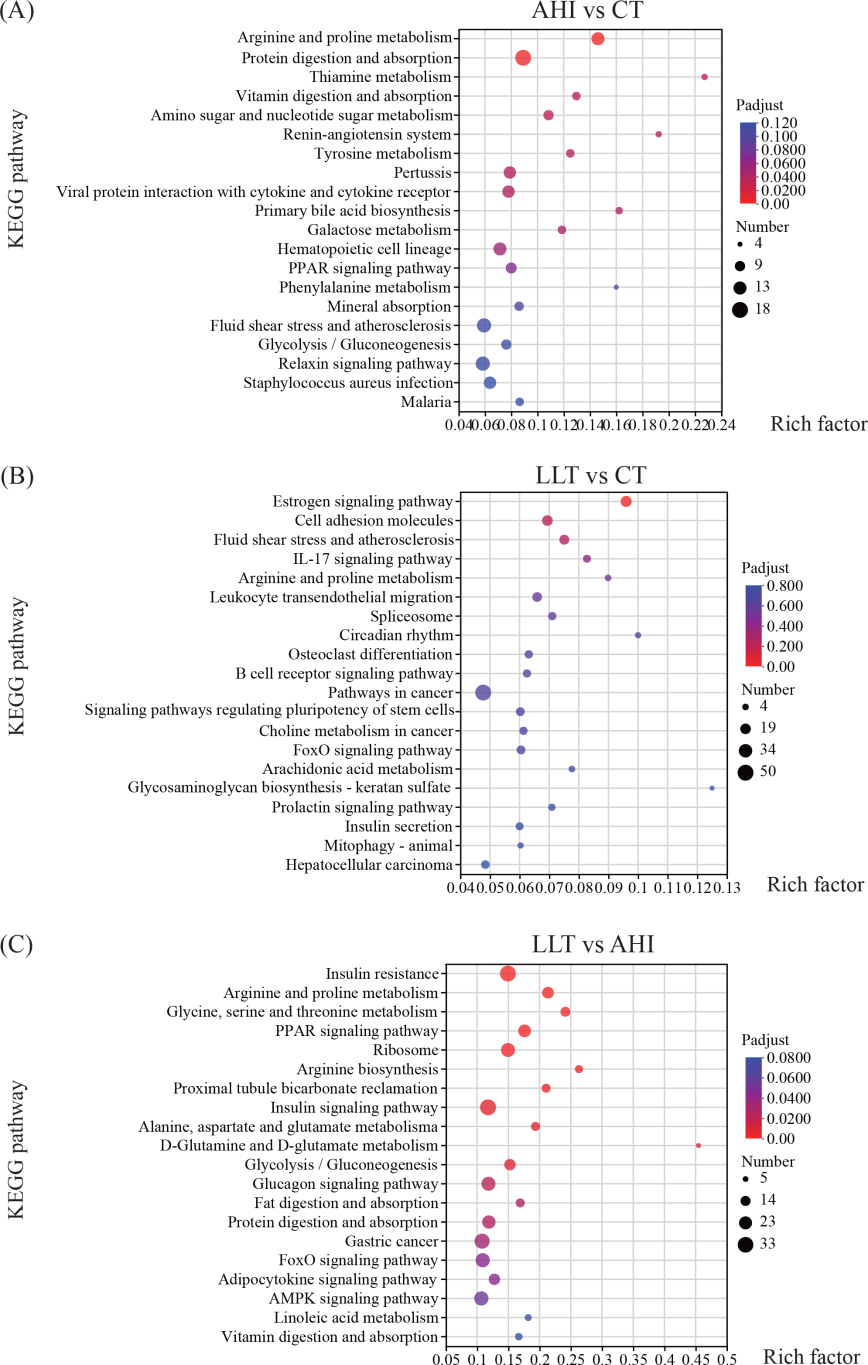
Bubble diagrams of the top 20 enriched KEGG pathways among different groups. (**A**) AHI vs CT. (**B**) LLT vs CT. (**C**). LLT vs AHI.

**Fig 7:**
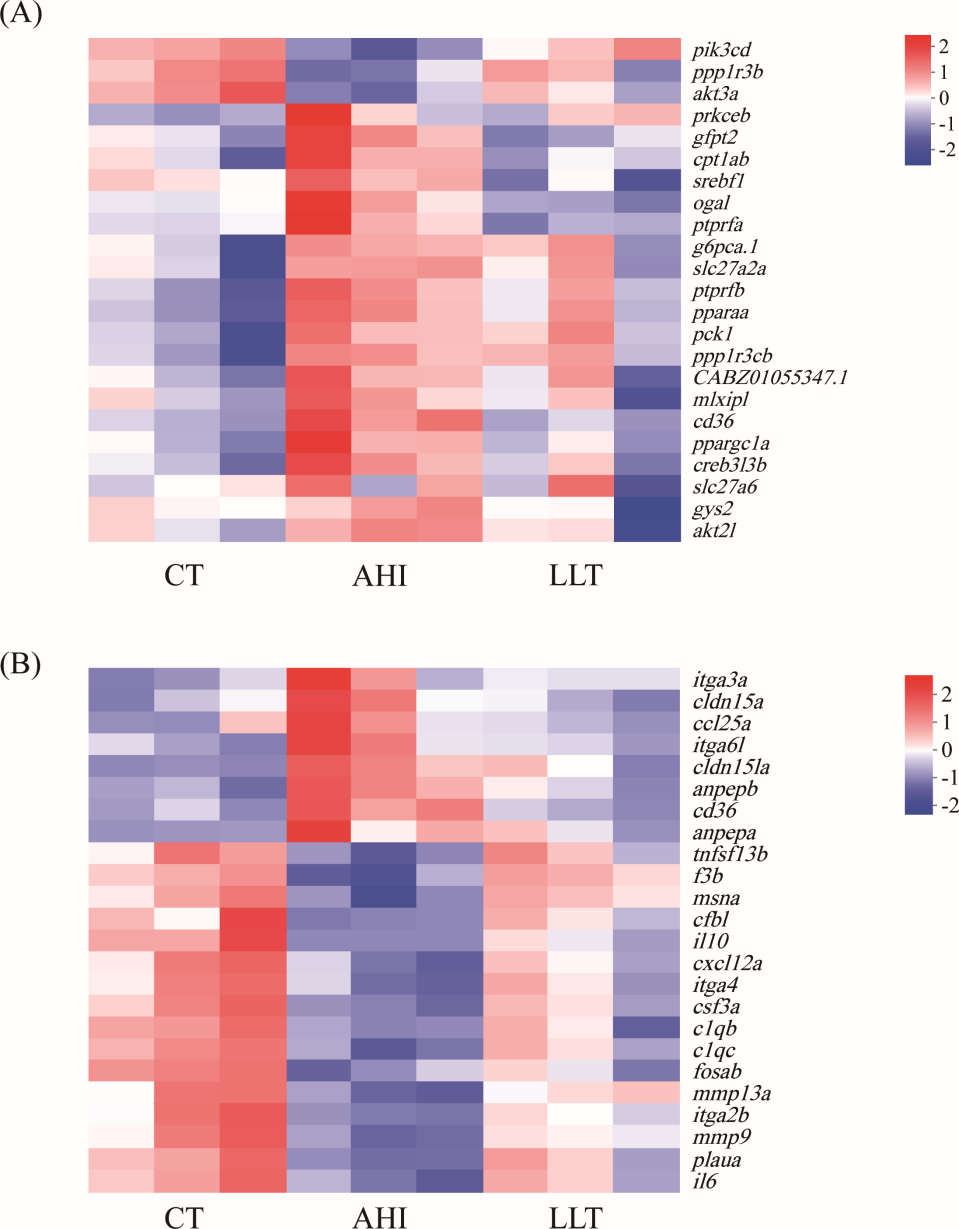
Heatmap of DEGs in insulin resistance pathway (**A**) and immune-related pathway (**B**).

A total of 778 DEGs in the LLT vs CT group were divided into 296 pathways. There were 20 pathways related to the immune system, including leukocyte transendothelial migration (18 genes, 2.31%), B-cell receptor signaling pathway (13 genes, 1.67%), IL-17 signaling pathway (12 genes, 1.54%), platelet activation (11 genes, 1.41%), and chemokine signaling pathway (11 genes, 1.41%). A total of 1,333 DEGs in the LLT vs AHI group were assigned to 316 pathways. Insulin resistance, insulin signaling pathway, and peroxisome proliferators-activated receptors (PPAR) signaling pathway were significantly enriched. The 20 most enriched pathways of each group are shown in Fig. 6. Compared to the AHI group, the LLT group showed expression levels of immune-related genes and insulin resistance genes that were more similar to those observed in the CT group (Fig. 7; Fig. S1).

### Validation of differently expressional genes by qRT-PCR

**Fig 8:**
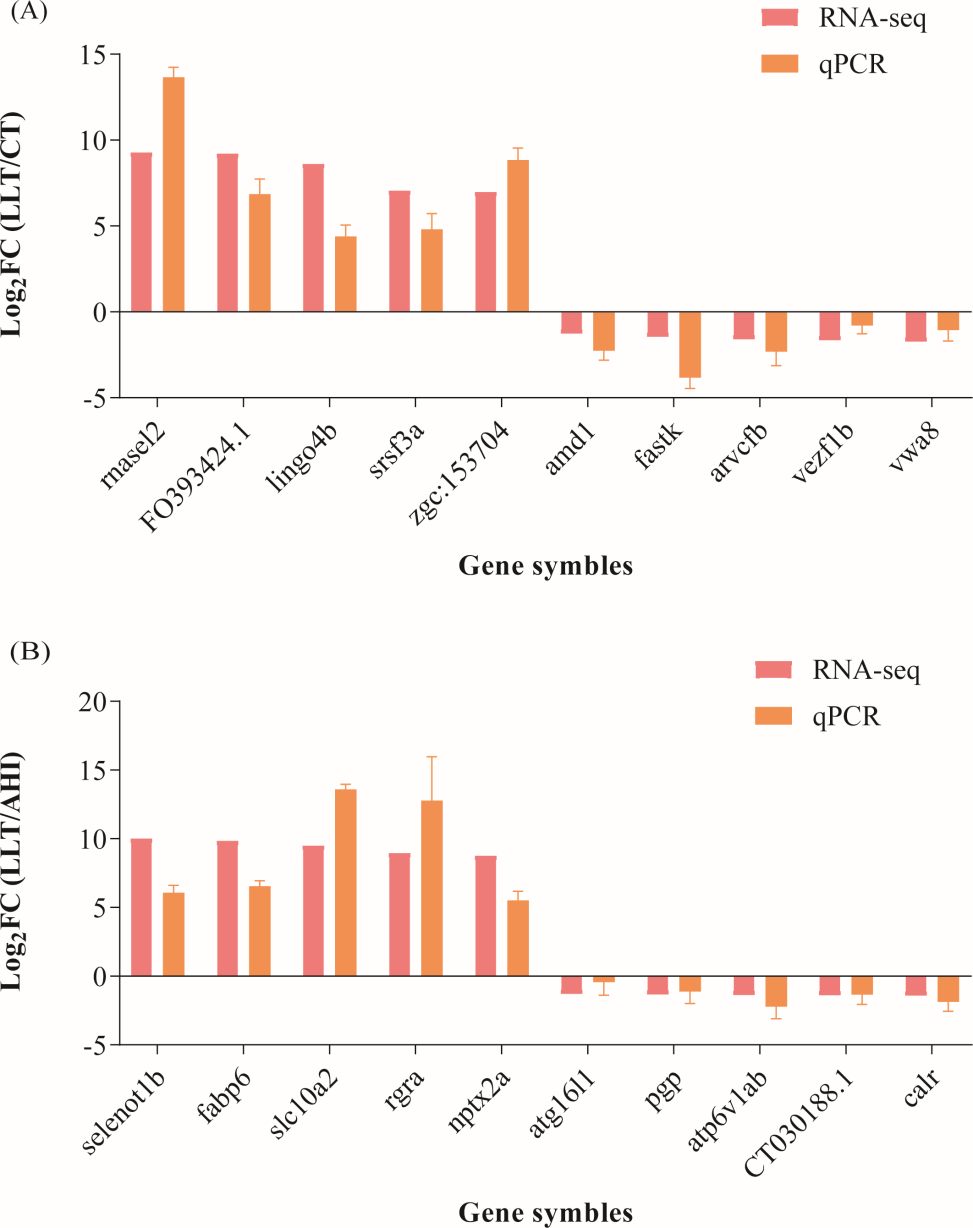
Validation of RNA-seq results by qPCR. (**A**) LLT vs CT. (**B**). LLT vs AHI.

Twenty genes with significant differences and immune correlation were selected for qPCR validation. The qRT-PCR primer sequences are shown in Table S4. The validation results for these 20 genes were consistent with the trend of differential expression profiles from RNA-seq, indicating that the RNA-seq results were highly accurate (Fig. 8).

## DISCUSSION

Transcriptome analysis is an important approach to expand the understanding of gene function, expression, and regulation. Molecular research on the immune response of fish to pathogenic bacteria has been very common (4, 7, 17, 33). Many probiotics have been shown to inhibit the growth of pathogenic bacteria and improve the immunity of fish (34–36); these past studies are consistent with the results of this study. Few studies have investigated the mechanism of immune response by analyzing the transcriptome of treated fish. The spleen is an immune organ that functions for the storage, production, and maturation of various granulocytes; it provides sufficient blood and a large number of immune cells for the fish (37–39). Here, the spleen transcriptome of zebrafish was analyzed across infection and treatment groups. More than 95% of the total identified genes were annotated. This functional information provides data on the immune response in a new perspective that enables exploration of fish infection protection mechanisms.

### The strongest immune response occurred 12 hours after infection

Clinical signs of infection in fish are bleeding on the body surface, redness and swelling of the anus, local tissue necrosis, and swelling of the abdomen (40). During the immune response period, DEGs were identified as related to the pattern-recognition receptors such as Toll-like receptors (TLRs) and Nod-like receptors (NLRs). TLRs are located on the surface of immune cells, such as macrophages and dendritic cells, as well as in intracellular compartments, such as endosomes. The duration of the immune response is different among different infected fish (4, 33, 41). After infection with *A. hydrophila*, the expression levels of IL are highest at 12 hours (42). After infection with *A. hydrophila*, the expression levels of antimicrobial peptide genes first increased and then returned to normal levels in Mandarin fish (*Siniperca chuatsi*) (43). In this study, the zebrafish in the AHI group showed a large number of deaths after 12 hours of infection. At the later stage after infection, the gene *IL6* belongs to the TLR and NLR pathway and was significantly downregulated after challenge with *A. hydrophila,* while there were no significant differences between the CT and LLT groups. It is not surprising that *IL6* is significantly downregulated at 48 hours, as the strongest immune response occurred 12 hours after infection. IL6 is a soluble mediator that has a variety of effects on inflammation, immune response, and hematopoiesis (44, 45). We speculated that once the strongest immune response has ended, the body initiated a negative regulatory mechanism to avoid adverse effects caused by excessive activation of immune-related pathways. At the later stage after infection, the negative regulatory effect has been excessive. The protein encoded by these genes is increasing and will eventually return to normal levels. The expression of these genes was not significantly different between the CT and LLT groups. The only significantly enriched pathway between the CT and LLT groups was the estrogen signaling pathway. It indicated that pro-inflammatory factors had returned to normal levels in the LLT group. Zebrafish treated with *L. lactis* KUST48 completed the immune response more quickly.

### Metabolic disorders caused by *A. hydrophila*

DEGs in the AHI vs CT group were significantly enriched in many metabolic pathways such as arginine and proline, thiamine, amino and nucleotide sugar, tyrosine, and galactose metabolism. This indicated that *A. hydrophila* challenge severely affects the normal metabolism of zebrafish. Arginine and its metabolites, along with thiamine, are important for reproduction, growth, development, and immunity (46, 47). Arginine can regulate energy homeostasis via modulating the adenosine 5′-monophosphate-activated protein kinase pathway (48). Polyamines, a kind of arginine metabolic product, promote cell growth and differentiation. Adding appropriate arginine to feed can improve growth performance and free amino acid concentrations in hemolymph and immune response through Nrf2 and TLR/nuclear factor kappa-B(NF-κB) signaling (49). Amino acids are considered essential for the regulation of immune response. They activate T lymphocytes, B lymphocytes, natural killer cells, and macrophages. Moreover, they regulate the production of antibodies, cytokines, and other cytotoxic substances and improve gene expression and lymphocyte proliferation (50).

### Insulin resistance pathway

Insulin resistance was the most enriched DEG immune-related pathway between LLT and AHI in zebrafish. Research has shown that insulin can promote the proliferation and differentiation of lymphocytes, which enhances the number and function of immune cells (51). In addition, it can also regulate the activation and function of immune cells. In our study, the expression of *ppargc1a*, *ogal*, *cd36*, *srebf1*, *creb3l3b*, *gfpt2*, *ptprfa*, and *cpt1ab* was significantly upregulated after *A. hydrophila* attack. *Ppargc1a*, *srebf1*, and *creb3l3b* are transcription factors involved in the regulation of protein tyrosine phosphatases (PTPs) and insulin receptor substrate-1 (IRS-1). PTPs is a protein tyrosine phosphatase that can inhibit insulin signaling by dephosphorylating IRS-1 (52). The upregulation of PTPs after infection may increase the dephosphorylation of IRS-1 and further reduce the effect of insulin signaling. Research has shown that Chinese bayberry leaves proanthocyanidins (53) and quercitrin (54) alleviate insulin resistance by activating the phosphoinositide-3-kinase (PI3K) pathway. This may be an important reason for the upregulation of PI3K. The upregulation of PI3K expression may compensate for the loss of insulin signaling pathway and promote the regulation of cell growth and metabolism by increasing PI3K activity. In our study, *L. lactis* has been shown to possess inhibitory effects on the growth of *A. hydrophila* and to attenuate the associated inflammatory response. The insulin resistance in treated zebrafish was alleviated. This is evidenced by the significant downregulation of these genes between LLT and AHI groups, with no significant difference compared to the CT group. The administration of *L. lactis* has the potential to alleviate the metabolic disorder and insulin resistance induced by *A. hydrophila* (40).

### Conclusion

The impact of *L. lactis* KUST48 on the transcription profile of *A. hydrophila*-infected zebrafish spleen was conducted. This study helps to strengthen our understanding of the pathogenic mechanisms of *A. hydrophila* in zebrafish and provide a valuable reference for the molecular mechanisms of probiotic therapy. Additionally, our findings can contribute to related studies on other economically important aquatic species. The transcriptomic data suggest that *L. lactis* may have a potential role in the treatment of AHIs in aquaculture, serving as a potential alternative to antibiotics.

## Data Availability

All clean data generated in this study were submitted to the NCBI Sequence Read Archive (SRA) database under accession numbers SRR22163682 to SRR22163690.

## SUPPLEMENTAL MATERIAL

Figure S1 spectrum.03927-23-s0001.docx The number of DEGs in different pathways.

Table S1 spectrum.03927-23-s0002.xls GO functional classification of DEGs.

Table S2 spectrum.03927-23-s0003.xls KEGG functional annotation and classification of DEGs.

Table S3 spectrum.03927-23-s0004.xls Enriched KEGG pathways.

Table S4 spectrum.03927-23-s0005.xls qRT-PCR primer sequences.

ASM does not own the copyrights to Supplemental Material that may be linked to, or accessed through, an article. The authors have granted ASM a non-exclusive, world-wide license to publish the Supplemental Material files. Please contact the corresponding author directly for reuse.
