# Supplementary material for: The impact of Lactococcus lactis KUST48 on the transcription profile of Aeromonas hydrophila-infected zebrafish spleen
Source: Microbiol Spectr. 2024 Mar 5;12(4):e03927-23. doi: 10.1128/spectrum.03927-23 (PMC10986548; doi:10.1128/spectrum.03927-23)
Supplement: Figure S1 — The number of DEGs in different pathways. [file spectrum.03927-23-s0001.docx]

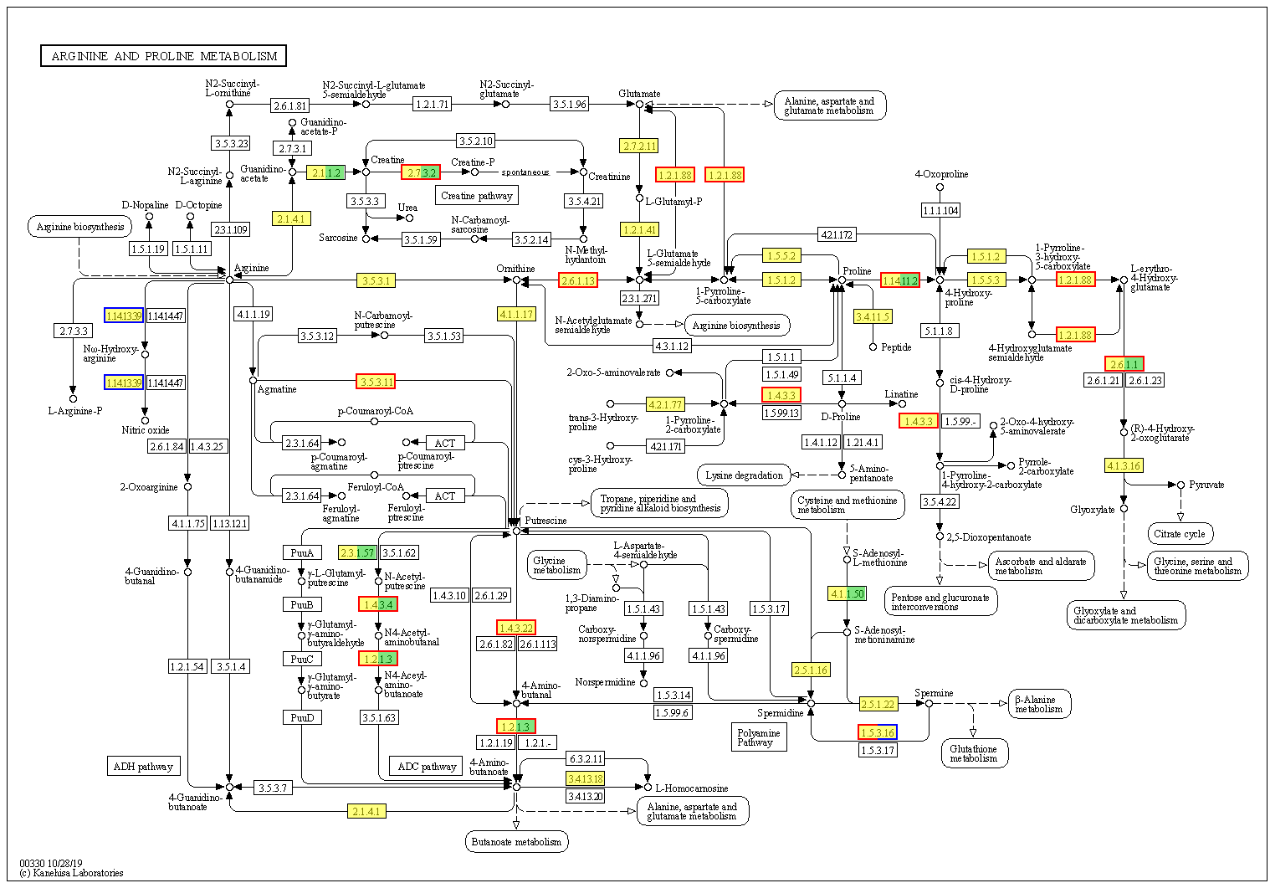

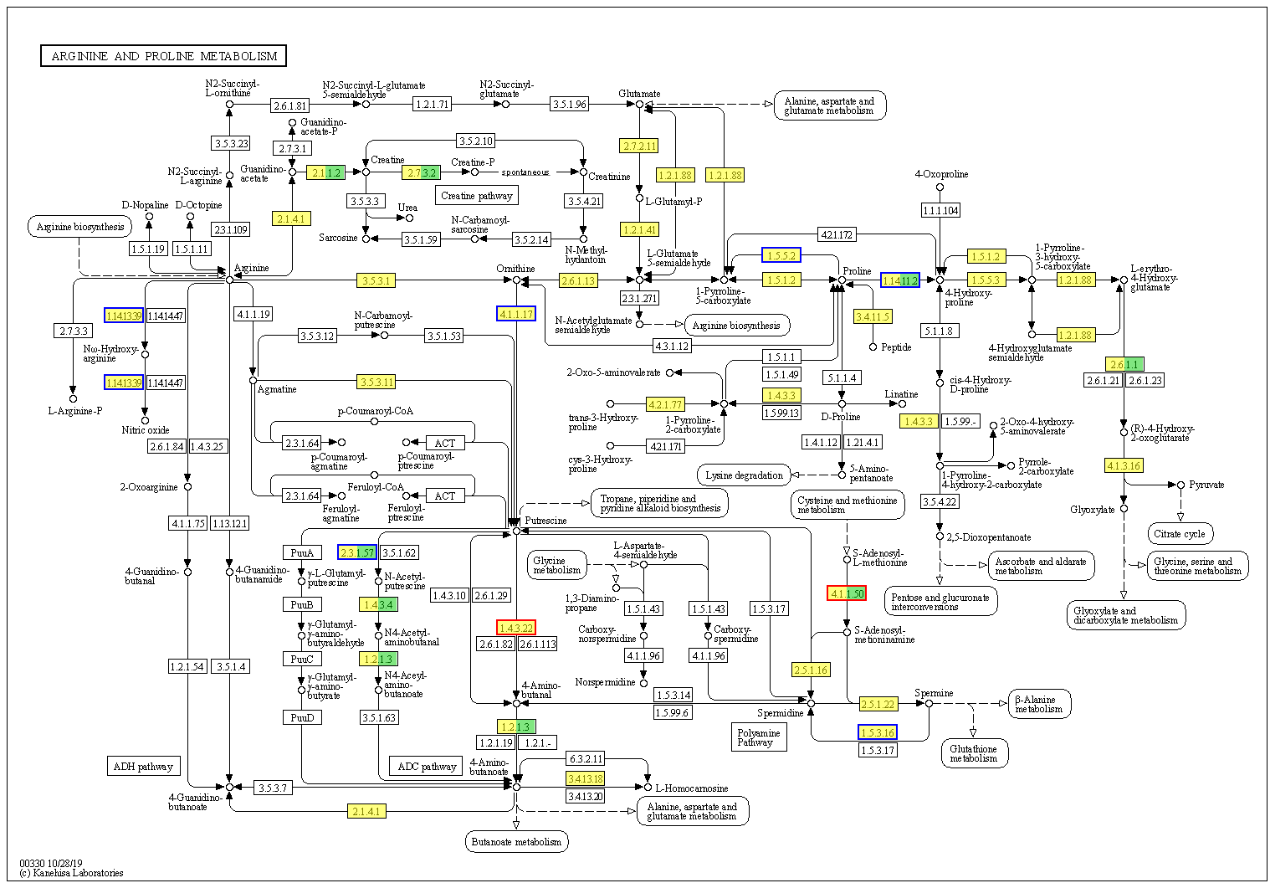


(B)

(A)


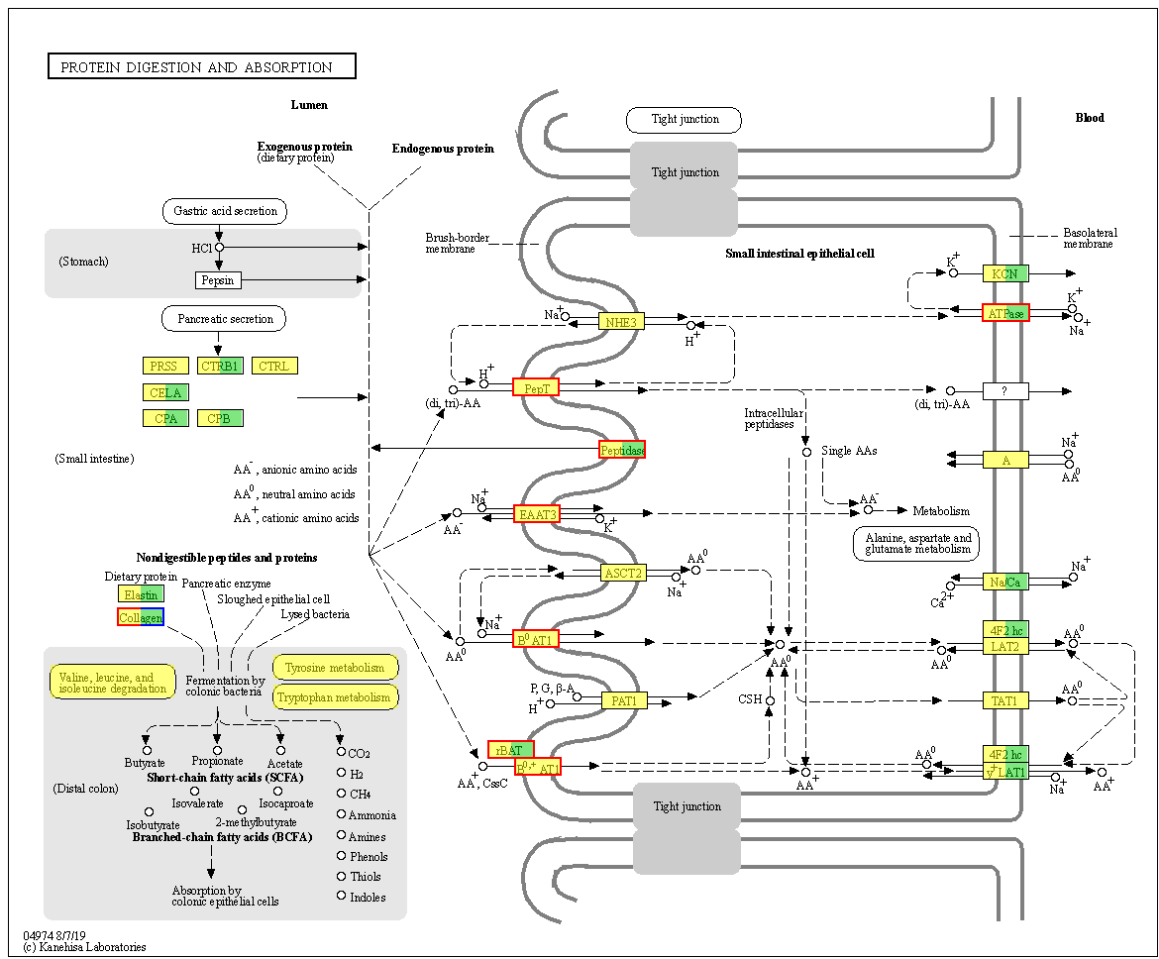

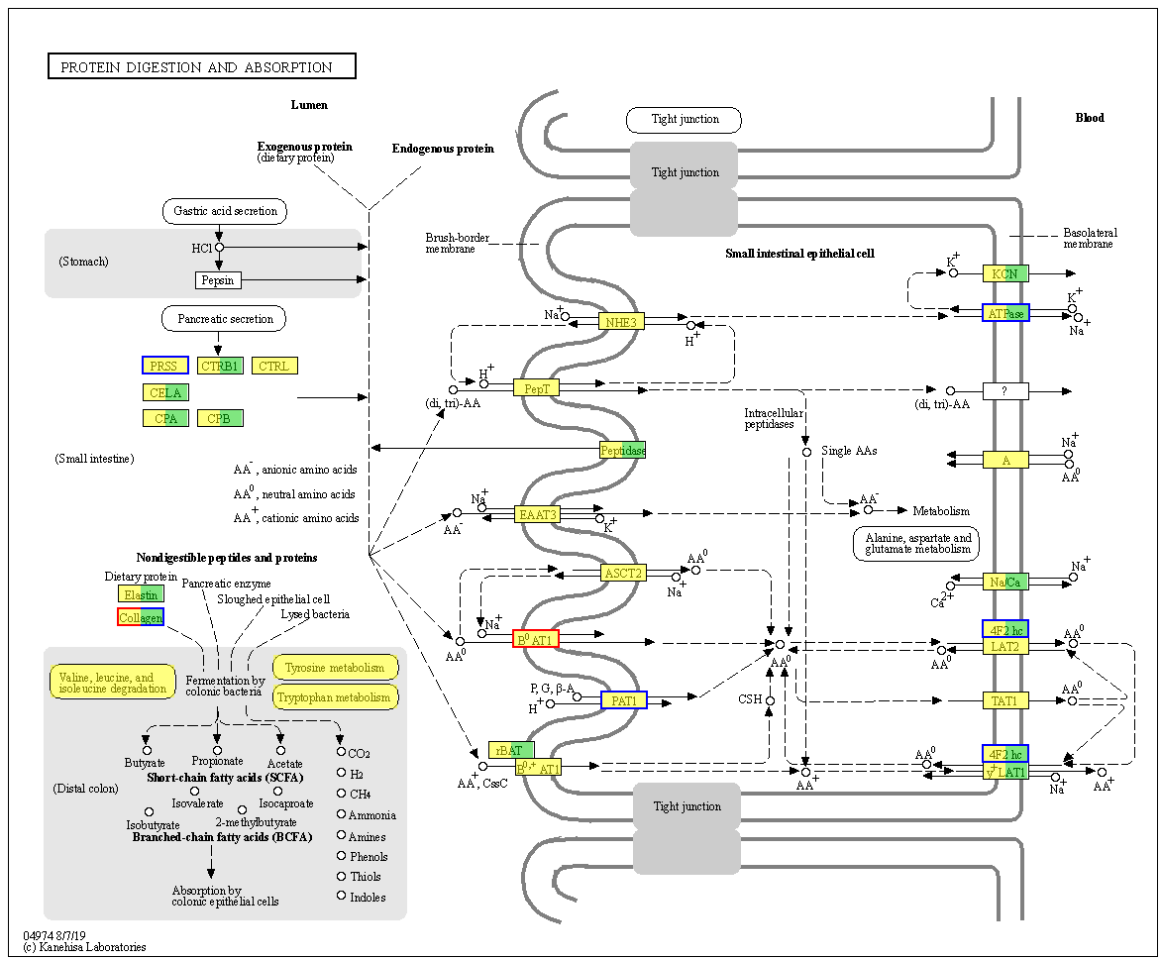

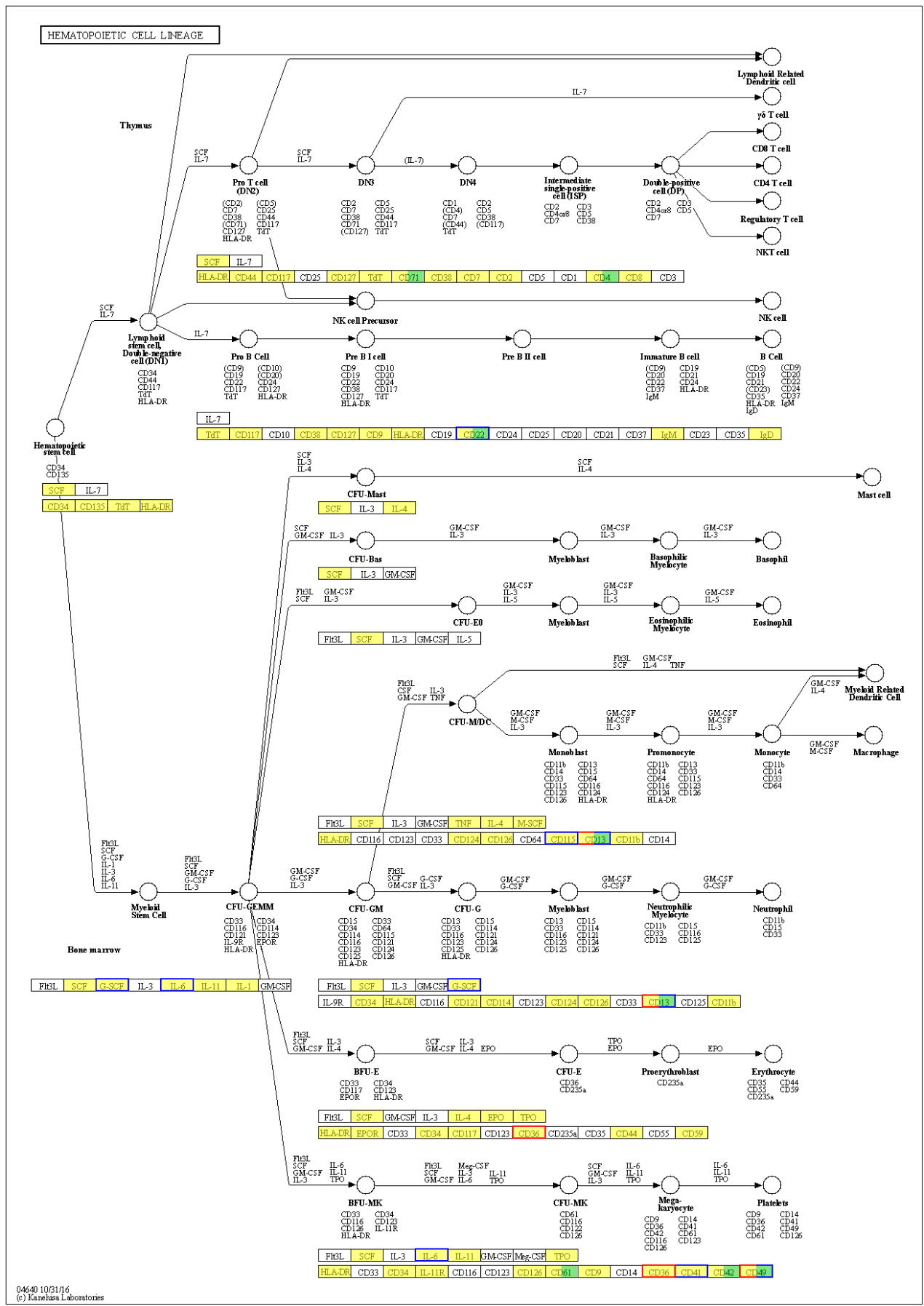

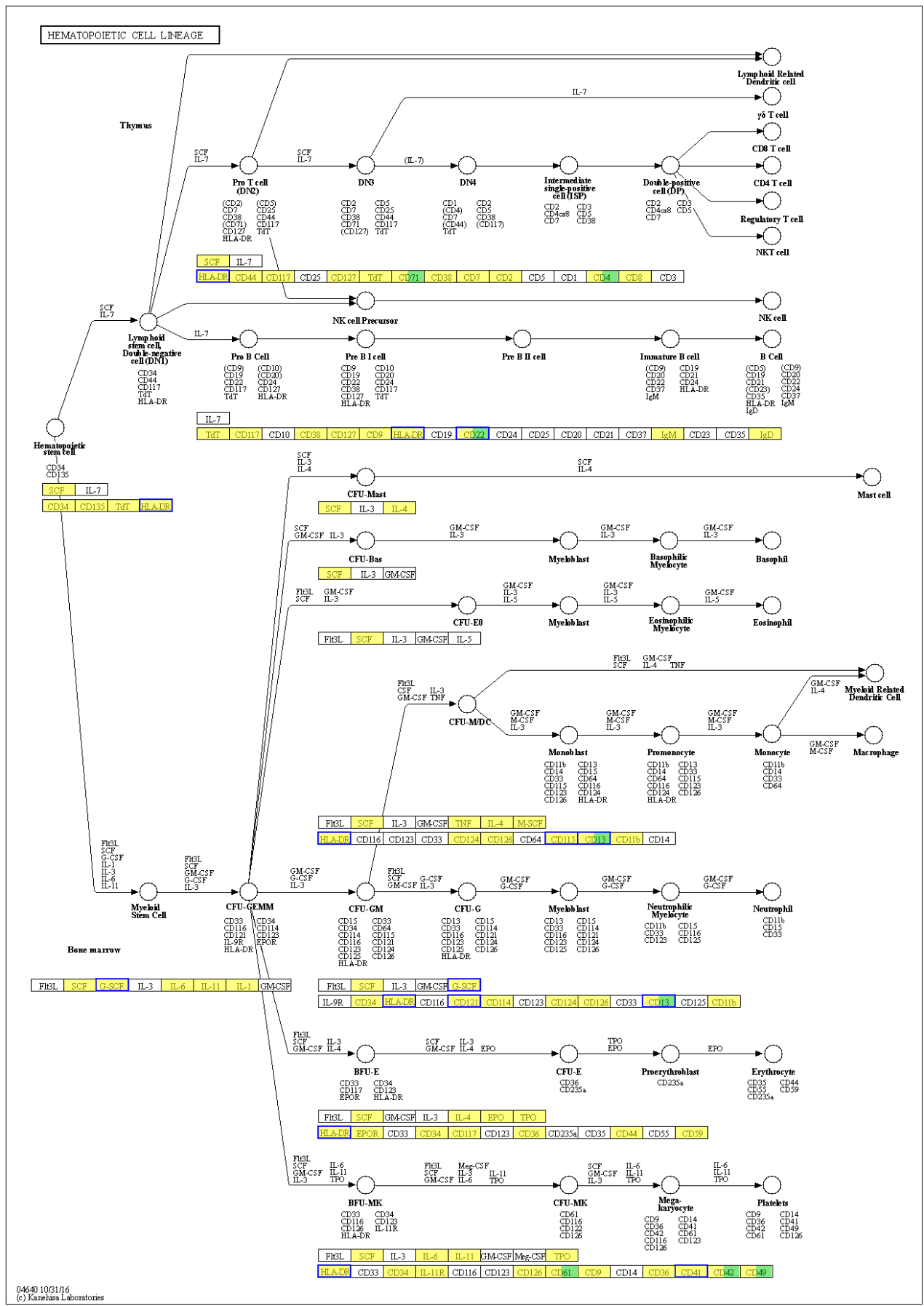


(D)

(C)

(E)

(F)


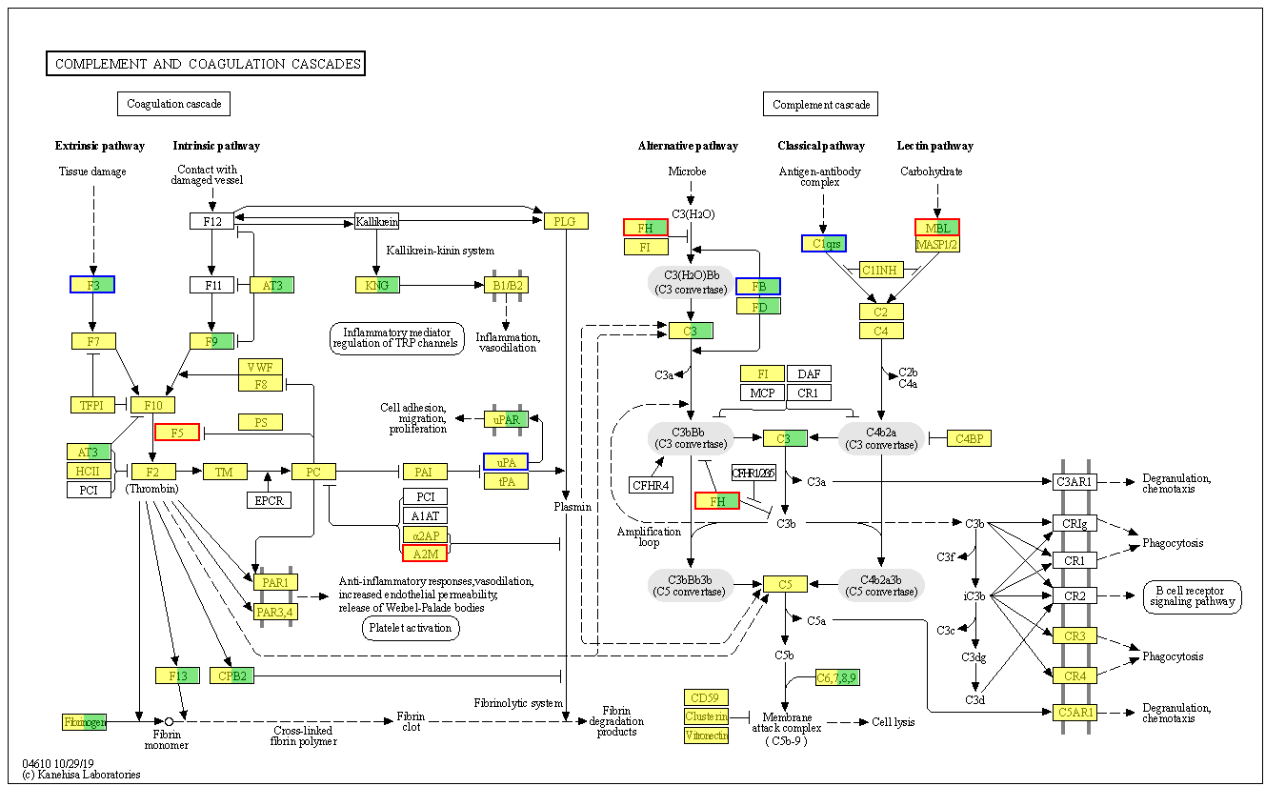

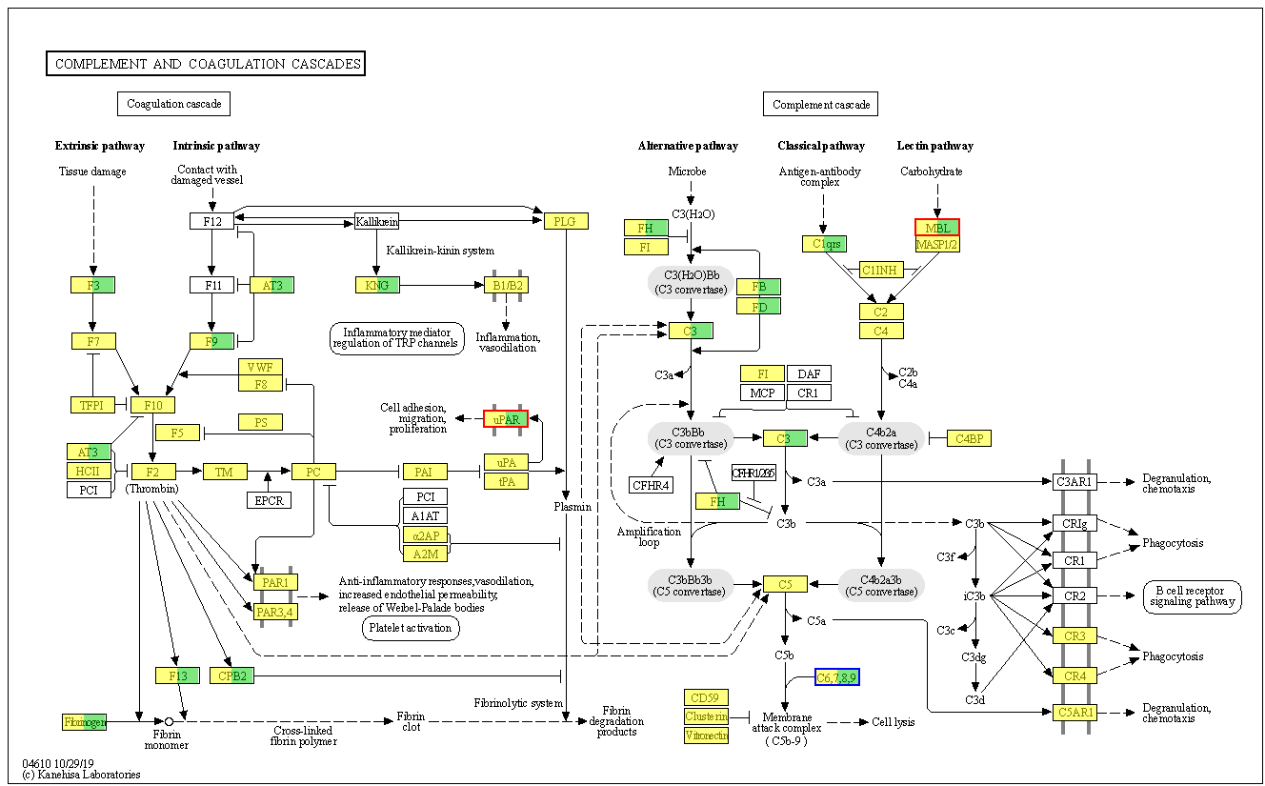


(H)

(G)

Fig. S1 The number of DEGs in different pathways. The red box represents up-regulated genes, while the blue box represents down-regulated genes. (A) DEGs between AHI and CT group in Arginine and proline metabolism pathway. (B) DEGs between LLT and CT group in Arginine and proline metabolism pathway. (C) DEGs between AHI and CT group in Protein digestion and absorption pathway. (D) DEGs between LLT and CT group in Protein digestion and absorption pathway. (E) DEGs between AHI and CT group in Hematopoietic cell lineage pathway. (F) DEGs between LLT and CT group in Hematopoietic cell lineage pathway. (G) DEGs between AHI and CT group in Complement and coagulation cascades pathway. (H) DEGs between LLT and CT group in Complement and coagulation cascades pathway.
